# A multicenter clinical evaluation of polymerase chain reaction coupled with quantum dot fluorescence analysis in pathogen detection for patients with suspected bloodstream infections

**DOI:** 10.1128/spectrum.03845-25

**Published:** 2026-07-21

**Authors:** Guangzhi Du, Wenjie Yang, Bangxing Lin, Xinghan Huang, Junjie Lao, Yueming Chen, Xueyan Dong, Lihui Xu, Xianjun Wang

**Affiliations:** 1 The Fourth School of Clinical Medicine, Zhejiang Chinese Medical University 70571, Hangzhou, China; 2 Department of Clinical Laboratory, Affiliated Hangzhou First People's Hospital, School of Medicine, Westlake University 74630, Hangzhou, China; Nationwide Children's Hospital, Columbus, Ohio, USA

**Keywords:** PCR-QDFA, molecular diagnostics, rapid pathogens, bloodstream infections

## Abstract

Bloodstream infections remain a serious clinical challenge and are associated with substantial morbidity and mortality, particularly in critically ill patients. Early identification of the causative pathogens is essential for timely antimicrobial therapy, yet conventional blood culture methods often require prolonged incubation and additional processing time before results become available. In this study, we evaluated the clinical performance of polymerase chain reaction coupled with quantum dot fluorescence analysis (PCR-QDFA) for pathogen detection in patients with suspected bloodstream infections. The assay showed strong agreement with routine culture-based identification while providing results in a much shorter time. Its ability to simultaneously detect multiple common bloodstream pathogens makes it suitable for use in routine diagnostic laboratories. Rapid pathogen identification through PCR-QDFA may help clinicians make earlier treatment decisions, support more appropriate antimicrobial use, and improve the overall management of bloodstream infections.

## INTRODUCTION

Bloodstream infection (BSI) is associated with high morbidity and mortality worldwide. Globally, BSI ranks among the top seven causes of death in North America and Europe (1). Early initiation of appropriate antibiotic treatment for BSI, especially within the first hour after clinical diagnosis, is crucial for reducing morbidity and mortality (2). BSI patients are at a high risk of developing sepsis. BSI accounts for approximately 40% of sepsis and septic shock cases overall, including both community-acquired and hospital-acquired infections, and about 20% of intensive care unit-acquired cases (3). For patients at risk of septic shock, timely administration of antibiotics is essential; a delay in antimicrobial treatment can lead to worse outcomes: for every 6-h delay in treatment, the survival rate decreases by more than 7% (2, 4). Due to factors such as increased antibiotic resistance, increased use of medical devices, and an increase in immunocompromised individuals, the incidence of BSI is on the rise each year. Rapid and accurate bacterial identification methods are needed.

A fast and accurate approach for bloodstream infection diagnosis leads to better patient outcomes (5). However, the standard method using microbiological culture takes an average of 4 days. After blood cultures become positive, several steps are needed. These steps include Gram staining, culturing on plates, and identifying microbes with mass spectrometry. Completing these steps typically requires 24–72 h to get final results (6). Additionally, this traditional process faces challenges. It struggles with microorganisms that grow slowly, live inside cells, or have demanding growth requirements. Results are also less reliable for patients already on antimicrobial drugs.

Molecular biological methods can be directly applied to whole blood samples, saving the time required for culture. This is particularly advantageous when the bacterial growth in the sample is slow or the bacterial load is low. Polymerase chain reaction coupled with quantum dot fluorescence analysis (PCR-QDFA) is a new molecular diagnostic technique. It involves designing oligonucleotide probes with amino groups at the 3′ end to specifically recognize gene fragments of various pathogenic bacteria, which are then fixed at specific positions on a nylon membrane to create a detection membrane strip. The 5′ end of the downstream primer is labeled with biotin. The PCR amplification products labeled with biotin are first hybridized with the probe molecules on the detection membrane strip and then bound to quantum dots conjugated with streptavidin through the biotin-streptavidin interaction. When the detection membrane strip is passed through a fluorescence detection instrument, the presence or absence of fluorescence signals at each site is observed to determine whether the probe has hybridized with the PCR product, thereby determining whether the sample contains the relevant pathogen.

PCR-QDFA is a recently developed molecular assay designed for rapid, multiplex identification of clinically relevant pathogens. Although early studies have suggested strong diagnostic potential, large-scale clinical validation remains limited, particularly in multicenter cohorts. This study aims to comprehensively evaluate the diagnostic performance of PCR-QDFA for detecting common bloodstream pathogens across four clinical institutions, using blood culture as the reference method and Sanger sequencing for discrepancy resolution.

## MATERIALS AND METHODS

### Sample size and justification

Clinical trials of nucleic acid amplification-based in vitro diagnostic reagents for pathogen detection generally require a large sample size, and in this study, a total of over 2,600 cases were included to ensure the robustness of the analysis. The investigational assay is designed to detect 29 pathogenic bacteria in human blood cultures. Generally, the number of positive samples should account for no less than 30% of the total sample size. However, considering the possibility of polymicrobial infections in clinical samples and the low or rare prevalence of certain target pathogens, the required number of positive samples was adjusted accordingly. Therefore, this clinical trial planned to enroll a minimum of 2,500 cases, including both blood culture-positive and negative samples, through competitive multicenter recruitment. The final enrollment depended on actual sample availability. Given that some bloodstream pathogens had a clinical detection rate of less than 1%, and to avoid delays in product registration and commercialization, the trial progress was adjusted based on the real-time detection rates of target pathogens during the study.

### Research samples

The blood culture samples included in this study were obtained from a mixed patient population. The cohort covered a wide age range, from neonates (including preterm infants) to elderly patients up to 100 years old, thus representing both pediatric and adult populations. Overall, the data set reflects a real-world clinical population rather than a specific high-risk or narrowly defined subgroup. Only samples tested by both the PCR-QDFA assay and blood culture using the same bottle medium were considered for analysis. A specimen was included when routine clinical testing had already defined it as positive or negative, its accompanying information was complete, the available volume reached at least 5 mL, and all handling steps followed the instructions provided by both manufacturers. Samples were excluded if they did not originate from blood, were collected in bottles designed for *Mycobacterium* culture, offered too little volume, lacked sufficient traceability, represented repeated submissions from the same patient, or had not been processed according to the required protocols. During the study, any specimen affected by operator mistakes, compromised reagent quality, or other circumstances judged inappropriate by the investigators was also removed from the data set.

### Blood culture

Blood cultures were performed using the BD BACTEC automated blood culture system. For each patient, two sets of blood culture samples were collected via venipuncture from different sites. Each set was inoculated into paired aerobic and anaerobic culture bottles (BD BACTEC PLUS Aerobic/F and Lytic/10 Anaerobic/F). For adult patients, 8–10 mL of blood was collected per bottle. All bottles were incubated in the BD BACTEC FX200 system for continuous monitoring. Upon a positive signal from the instrument, aliquots from both aerobic and anaerobic bottles were aseptically withdrawn using sterile syringes within a biosafety cabinet and subcultured onto Columbia blood agar plates and Sabouraud dextrose agar plates (with chocolate agar plates added when necessary). Plates inoculated from aerobic bottles were incubated at 35°C in a 5% CO_2_ atmosphere for 16–18 h. Plates inoculated from anaerobic bottles were incubated in parallel at 35°C under both 5% CO_2_ conditions and anaerobic conditions (using an anaerobic chamber or anaerobic gas-generating system). Single colonies were selected from the plates and suspended in sterile saline to prepare bacterial suspensions. The turbidity was adjusted using a densitometer to the McFarland standard required for the respective identification cards (0.5–0.63 McF for common bacteria, 1.8–2.2 McF for yeasts, and 2.7–3.3 McF for fastidious organisms). Species identification was then performed using the VITEK 2 Compact automated microbial identification system with the corresponding identification cards.

### PCR-QDFA

Specific PCR primer pairs were designed based on conserved sequences of each pathogen. The 5′ end of the reverse primers was labeled with biotin. These primers were used to amplify gene fragments of specific lengths. According to the sequence differences of different genes, oligonucleotide probes were designed based on the principle of complementary base pairing. All probes were labeled with an amino group at the 3′ end and immobilized on specific positions of a nylon membrane through chemical bonds to form detection strips. Each of the 29 target pathogens is detected using its own pair of pathogen-specific primers and corresponding probes, all designed to ensure high specificity at the sequence level. Due to the large number of targets included in the panel, amplification could not be performed in a single reaction tube. Therefore, the assay was divided into three parallel PCR reactions, each containing a subset of primers, to achieve multiplex amplification while maintaining amplification efficiency and specificity. The amplified products were hybridized to membrane-bound probes with predefined spatial positions. Test results were interpreted based on the presence or absence of fluorescent spots at each position, with each spot corresponding to a specific pathogen. To avoid false-negative results in clinical testing, an internal control was included. This was done by amplifying the human housekeeping gene β-globin using specific primers and detecting it with a specific probe. This study employed a blood pathogen nucleic acid detection kit. The kit was developed by Hangzhou Qianji Biotechnology Co., Ltd. It leverages the principle described above and is pending registration.

### Resolution of discordant results

The test results of PCR-QDFA and blood culture were subjected to statistical analysis using appropriate statistical methods. The consistency of the test results between PCR-QDFA and blood culture was assessed, and the accuracy of the test results of PCR-QDFA was evaluated. In case of inconsistent results between PCR-QDFA and blood culture, third-party Sanger sequencing was conducted for rechecking to objectively evaluate PCR-QDFA.

### Statistical methods

Statistical analysis was performed for all 29 pathogens detected by the investigational assay in this clinical trial to determine the positive agreement rate, negative agreement rate, overall agreement rate (each with 95% confidence intervals), and Kappa values. Based on these analytical results, the clinical trial institutions provided an authentic and reliable clinical performance evaluation of the product. The statistical analyses were performed by SPSS 31.0.

### Quality control methods

This is an *in vitro* diagnostic reagent. It does not make direct contact with the human body. Thus, it causes no harm to study subjects. During validation, all data were used solely for clinical trial evaluation purposes and were not used for patient diagnosis, thus avoiding potential medical disputes. Prior to the trial, the sponsor provided investigators with technical training to ensure proper implementation. Rigorous clinical trial monitoring was maintained throughout the study to guarantee data authenticity, completeness, and protocol compliance. Testing procedures strictly followed the reagent instructions and adhered to the trial site’s quality control system. All instruments underwent scheduled maintenance as required. Post-trial, original data were preserved intact for traceability and are subject to regulatory audits at any time.

### Sample distribution

Among 2,619 blood culture bottles, 2,195 positive bottles were identified through blood culture, and 2,195 suspected BSI pathogens were identified through blood culture (2,093 from monomicrobial blood culture bottles and 102 from polymicrobial ones). The three most prevalent gram-negative pathogens were *Escherichia coli* (*n* = 488, 22.2%), *Klebsiella pneumoniae* (*n* = 368, 16.8%), and *Pseudomonas aeruginosa* (*n* = 68, 3.10%). The three most common gram-positive pathogens were *Staphylococcus hominis* (*n* = 203, 9.25%), *Staphylococcus epidermidis* (*n* = 149, 6.8%), and *Staphylococcus aureus* (*n* = 131, 5.97%). The predominant fungal species was *Candida albicans* (*n* = 48, 2.2%) (Table 1). A total of 2,195/2,195 pathogens were identified from positive blood culture bottles that were included in the PCR-QDFA list, covering 100% of the clinical isolates during the study period. In addition, two pathogens on the PCR-QDFA list were not collected in this sample set, including *Haemophilus influenzae* and *Candida krusei* (Table 1).

**TABLE 1:** Overall performance of the PCR-QDFA assay in the identification of each microorganism from 2,619 blood culture bottles

	Blood culture/PCR-QDFA				Sensitivity (%)	Specificity (%)	PPV (%)	NPV (%)	Agreement rate (%)	Kappa value	*P value*
Pathogens	+/+^*a*^	+/−^*b*^	−/+^*c*^	−/−^*d*^							
Pathogens on the PCR-QDFA list											
Gram negative											
*Acinetobacter baumannii*	67	0	13	2,539	100	99.49	83.75	100	99.5	0.91	<0.005
*Burkholderia cepacia*	20	0	1	2,598	100	99.96	95.24	100	99.96	0.98	<0.005
*Klebsiella aerogenes*	28	0	1	2,590	100	99.96	96.55	100	99.96	0.98	<0.005
*Enterobacter cloacae*	57	0	14	2,548	100	99.45	80.28	100	99.47	0.89	<0.005
*Escherichia coli*	488	0	32	2,099	100	98.5	93.85	100	98.78	0.96	<0.005
*Klebsiella oxytoca*	18	0	1	2,600	100	99.96	94.74	100	99.96	0.97	<0.005
*Klebsiella pneumoniae*	368	0	17	2,234	100	99.24	95.58	100	99.35	0.97	<0.005
*Proteus vulgaris*	1	0	0	2,618	100	100	100	100	100	1	<0.005
*Proteus mirabilis*	19	0	5	2,595	100	99.81	79.17	100	99.81	0.88	<0.005
*Pseudomonas aeruginosa*	68	0	2	2,549	100	99.92	97.14	100	99.92	0.99	<0.005
*Serratia marcescens*	21	0	3	2,595	100	99.88	87.5	100	99.89	0.93	<0.005
*Stenotrophomonas maltophilia*	29	0	4	2,586	100	99.85	87.88	100	99.85	0.93	<0.005
*Haemophilus influenzae*	0	0	0	2,619	n.a.	100	n.a.	100	100	n.a.	n.a.
Total	1,184	0	93	1,342	100	93.52	92.72	100	96.45	0.93	<0.005
Gram positive											
*Enterococcus faecalis*	79	0	5	2,535	100	99.8	94.05	100	99.81	0.97	<0.005
*Enterococcus faecium*	124	0	10	2,485	100	99.6	92.54	100	99.62	0.96	<0.005
*Streptococcus agalactiae*	18	0	3	2,598	100	99.88	85.71	100	99.89	0.92	<0.005
*Staphylococcus aureus*	131	0	3	2,485	100	99.88	97.76	100	99.89	0.99	<0.005
*Staphylococcus epidermidis*	149	0	17	2,453	100	99.31	89.76	100	99.35	0.94	<0.005
*Staphylococcus haemolyticus*	61	0	6	2,552	100	99.77	91.04	100	99.77	0.95	<0.005
*Staphylococcus hominis*	203	0	8	2,408	100	99.67	96.21	100	99.69	0.98	<0.005
*Viridans streptococci*	82	0	1	2,536	100	99.96	98.8	100	99.96	0.99	<0.005
*Streptococcus pneumoniae*	26	0	12	2,581	100	99.54	68.42	100	99.54	0.81	<0.005
*Streptococcus pyogenes*	2	0	0	2,617	100	100	100	100	100	1	<0.005
*Staphylococcus saprophyticus*	1	0	0	2,618	100	100	100	100	100	1	<0.005
Total	876	0	65	1,678	100	96.27	93.09	100	97.52	0.95	<0.005
Fungi											
*Candida albicans*	49	0	3	2,567	100	99.88	94.23	100	99.89	0.97	<0.005
*Candida glabrata*	19	0	1	2,599	100	99.96	95	100	99.96	0.97	<0.005
*Candida parapsilosis*	40	0	0	2,579	100	100	100	100	100	1	<0.005
*Candida tropicalis*	27	0	1	2,591	100	99.96	96.43	100	99.96	0.98	<0.005
*Candida krusei*	0	0	0	2,619	n.a.	100	n.a.	100	100	n.a.	n.a.
Total	135	0	5	2,479	100	99.8	96.43	100	99.81	0.98	<0.005

^*a*^ +/+ represents the number of episodes in which both blood culture and PCR-QDFA were positive.

^*b*^ +/− represents the number of episodes in which blood culture was positive, but PCR-QDFA was negative.

^*c*^ −/+ represents the number of episodes in which blood culture was negative, but PCR-QDFA was positive.

^*d*^ −/− represents the number of episodes in which both blood culture and PCR-QDFA were negative.

### Results of blood culture

**Fig 1:**
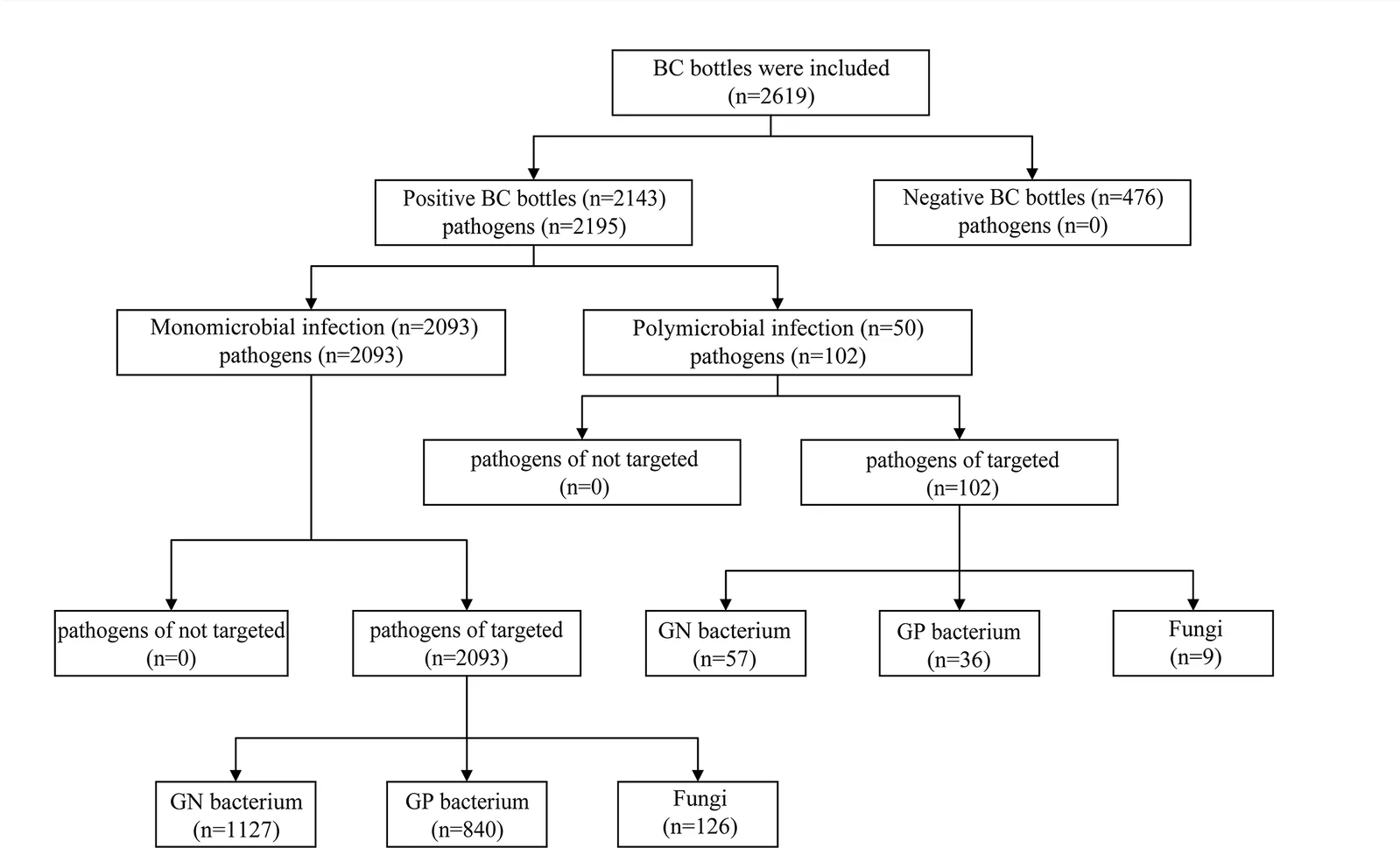
Flowchart of patient enrollment.

**Fig 2:**
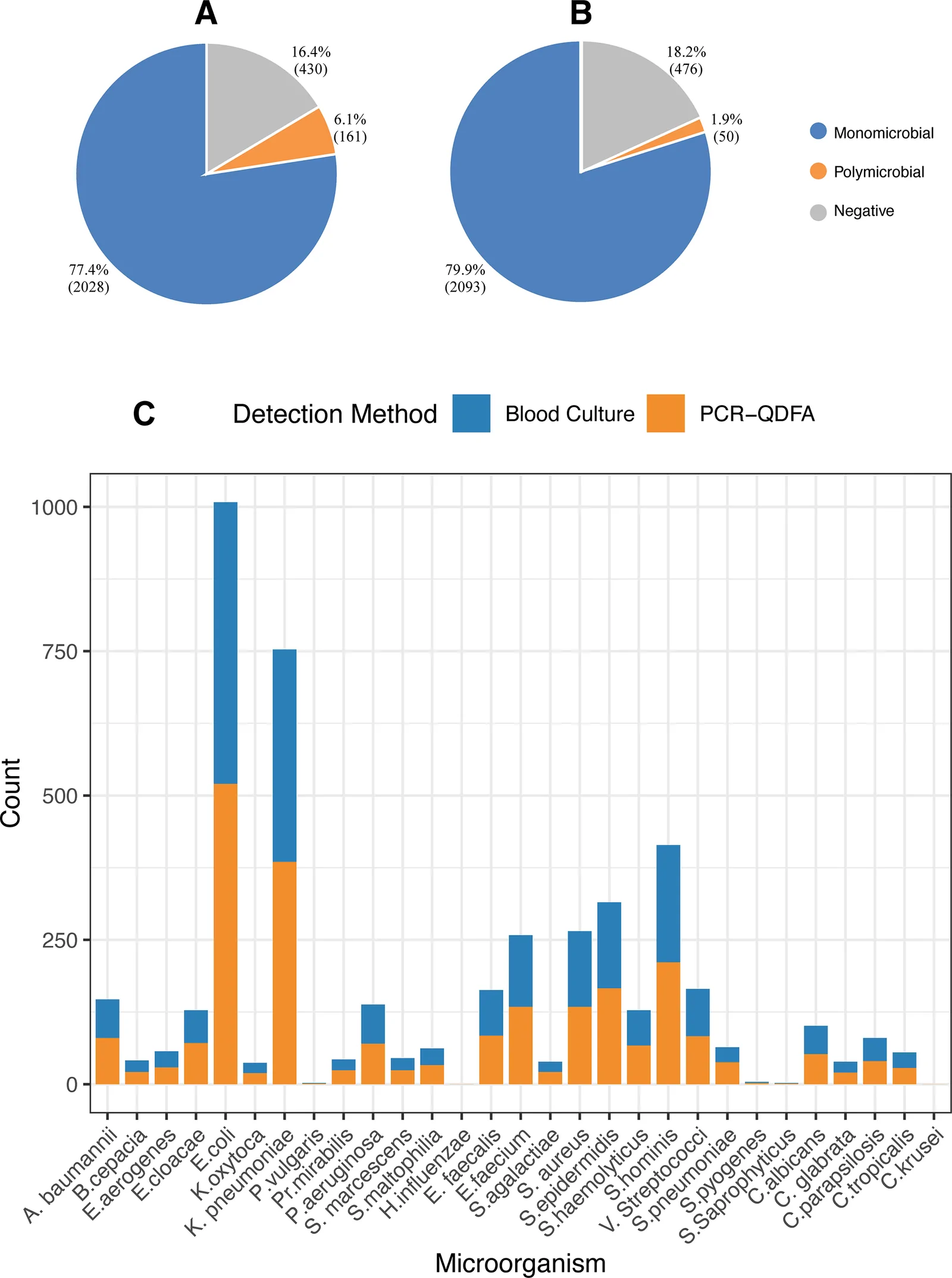
The detection result and distribution of pathogens identified by blood culture and PCR-QDFA. (A) PCR-QDFA detection results; (B) blood culture detection results; (C) distribution of pathogenic bacteria detected by the two methods.

Analysis of the 2,619 blood culture samples collected in this study revealed 2,143 positive cases and 476 negative cases. Among the positive samples, a total of 2,195 pathogenic microorganisms were identified. The majority (2,093 samples) showed monomicrobial growth, while polymicrobial growth was observed in 50 samples (Fig. 1 and 2B). Characterization of the isolates identified 1,184 gram-negative bacteria (representing 12 distinct species), 876 gram-positive bacteria (from 11 different species), and 135 fungi (comprising 4 species) (Fig. 1 and 2C).

### PCR-QDFA assay results

The PCR-QDFA assay initially detected 2,189 positive samples and 430 negative samples. Among the 2,189 positive samples, a total of 2,358 pathogenic microorganisms were identified (Table 1). Among the 2,189 positive samples, 2,028 cases showed monomicrobial detection and 161 cases showed polymicrobial detection (Fig. 2A). Within the 2,619 blood culture samples analyzed, PCR-QDFA identified 12 gram-negative bacterial species (1,277 isolates), 11 gram-positive bacterial species (941 isolates), and 4 fungal species (140 isolates) (Fig. 2C). Third-party Sanger sequencing was performed to re-examine these samples and revealed that one pathogen not on the PCR-QDFA target list was misidentified. Six cases of *Staphylococcus simulans* had been misidentified as *Staphylococcus haemolyticus*. Five of these misidentifications originated from polymicrobial samples, while one came from a negative sample. Cross-reactivity among staphylococci likely caused these errors. Additionally, one negative blood culture bottle was incorrectly reported as *Burkholderia cepacia* complex, a result subsequently attributed to procedural contamination.

### Detection performance of PCR-QDFA in monomicrobial growth

Blood culture identification and confirmation revealed that 2,093 out of 2,619 (79.92%) blood culture bottles exhibited monomicrobial growth. Among the 2,093 samples, 2,093 (100%) microorganisms were successfully identified by the PCR-QDFA assay using the results of blood culture as the reference (Table 2). All detected microorganisms were within the targets of PCR-QDFA. Notably, in 107/2,093 (7.1%) cases, the results of the PCR-QDFA assay were the same as those of blood culture, but it detected one to three additional pathogens than blood culture (Table 3). Third-party Sanger sequencing rechecked the 107 samples, and the PCR-QDFA assay verified the results of blood culture. This finding may indicate that the PCR-QDFA assay could help obtain an early indication of polymicrobial infection and help clinicians adopt the corresponding treatment strategy in a timely manner.

**TABLE 2:** Identification of bacteria and yeasts on the PCR-QDFA list from 2,093 monomicrobial blood culture bottles by the PCR-QDFA assay

	No. of samples			Sensitivity (%)
Identification	+/+^*a*^	+/−^*b*^	−/+^*c*^	
Gram negative				
*Acinetobacter baumannii*	62	0	7	100
*Burkholderia cepacia*	20	0	0	100
*K. aerogenes*	27	0	1	100
*Enterobacter cloacae*	54	0	12	100
*E. coli*	471	0	19	100
*Klebsiella oxytoca*	16	0	0	100
*K. pneumoniae*	346	0	14	100
*Proteus vulgaris*	1	0	0	100
*Proteus mirabilis*	16	0	4	100
*P. aeruginosa*	65	0	1	100
*Serratia marcescens*	21	0	2	100
*Stenotrophomonas maltophilia*	28	0	4	100
*H. influenzae*	0	0	0	n.a.
Total	1,127	0	64	100
Gram positive				
*Enterococcus faecalis*	69	0	3	100
*Enterococcus faecium*	106	0	7	100
*Streptococcus agalactiae*	18	0	1	100
*S. aureus*	130	0	1	100
*S. epidermidis*	147	0	10	100
*S. haemolyticus*	60	0	5	100
*S. hominis*	201	0	3	100
*Viridans streptococci*	80	0	1	100
*Streptococcus pneumoniae*	26	0	11	100
*Streptococcus pyogenes*	2	0	0	100
*Staphylococcus saprophyticus*	1	0	0	100
Total	840	0	42	100
Fungi				
*C. albicans*	43	0	2	100
*Candida glabrata*	18	0	1	100
*Candida parapsilosis*	40	0	0	100
*Candida tropicalis*	25	0	1	100
*C. krusei*	0	0	0	n.a.
Total	126	0	4	100
Total pathogens	2,093	0	110	100

^*a*^ Represents the number of episodes in which both blood culture and PCR-QDFA were positive.

^*b*^ Represents the number of episodes in which blood culture was positive, but PCR-QDFA was negative.

^*c*^ Represents the number of episodes in which blood culture was negative, but PCR-QDFA was positive.

**TABLE 3:** Discordant results of microorganisms on the PCR-QDFA list between blood culture and PCR-QDFA

Blood culture	PCR-QDFA			
	Pathogen 1	Pathogen 2	Pathogen 3	Pathogen 4
*S. hominis*	*S. hominis*	*K. pneumoniae*	–^*a*^	–
*K. pneumoniae*	*K. pneumoniae*	*A. baumannii*	–	–
*E. coli*	*E. coli*	*E. cloacae*	–	–
*E. coli*	*E. coli*	*E. cloacae*	–	–
*E. coli*	*E. coli*	*K. pneumoniae*	–	–
*S. epidermidis*	*S. epidermidis*	*K. pneumoniae*	–	–
*S. hominis*	*S. hominis*	*E. coli*	–	–
*S. epidermidis*	*S. epidermidis*	*E. coli*	–	–
*K. pneumoniae*	*K. pneumoniae*	*E. faecium*	–	–
*C. albicans*	*C. albicans*	*E. cloacae*	–	–
*S. maltophilia*	*S. maltophilia*	*S. pneumoniae*	–	–
*S. hominis*	*S. hominis*	*E. cloacae*	–	–
*E. coli*	*E. coli*	*S. haemolyticus*	–	–
*K. pneumoniae*	*K. pneumoniae*	*S. pneumoniae*	–	–
*K. pneumoniae*	*K. pneumoniae*	*S. pneumoniae*	–	–
*S. epidermidis*	*S. epidermidis*	*E. cloacae*	–	–
*K. pneumoniae*	*K. pneumoniae*	*S. hominis*	–	–
*E. coli*	*E. coli*	*C. albicans*	–	–
*S. marcescens*	*S. marcescens*	*S. haemolyticus*	–	–
*S. epidermidis*	*S. epidermidis*	*P. mirabilis*	–	–
*S. hominis*	*S. hominis*	*E. cloacae*	–	–
*S. aureus*	*S. aureus*	*S. hominis*	–	–
*E. coli*	*E. coli*	*P. mirabilis*	–	–
*K. pneumoniae*	*K. pneumoniae*	*S. epidermidis*	–	–
*E. faecalis*	*E. faecalis*	*S. epidermidis*	–	–
*A. baumannii*	*A. baumannii*	*K. pneumoniae*	–	–
*E. faecalis*	*E. faecalis*	*K. pneumoniae*	–	–
*S. marcescens*	*S. marcescens*	*E. cloacae*	–	–
*V. streptococci*	*V. streptococci*	*K. pneumoniae*	–	–
*E. faecium*	*E. faecium*	*E. faecalis*	–	–
*S. hominis*	*S. hominis*	*S. epidermidis*	–	–
*S. hominis*	*S. hominis*	*S. haemolyticus*	–	–
*A. baumannii*	*A. baumannii*	*V. streptococci*	–	–
*A. baumannii*	*A. baumannii*	*S. marcescens*	–	–
*S. hominis*	*S. hominis*	*S. haemolyticus*	–	–
*E. faecium*	*E. faecium*	*K. pneumoniae*	–	–
*S. hominis*	*S. hominis*	*S. epidermidis*	–	–
*P. aeruginosa*	*P. aeruginosa*	*C. tropicalis*	–	–
*E. faecium*	*E. faecium*	*S. maltophilia*	–	–
*K. pneumoniae*	*K. pneumoniae*	*S. epidermidis*	–	–
*P. aeruginosa*	*P. aeruginosa*	*A. baumannii*	–	–
*S. hominis*	*S. hominis*	*E. coli*	–	–
*S. hominis*	*S. hominis*	*E. coli*	–	–
*S. hominis*	*S. hominis*	*E. coli*	–	–
*E. coli*	*E. coli*	*K. pneumoniae*	–	–
*S. maltophilia*	*S. maltophilia*	*E. faecium*	–	–
*E. coli*	*E. coli*	*K. pneumoniae*	–	–
*S. epidermidis*	*S. epidermidis*	*E. coli*	–	–
*E. faecalis*	*E. faecalis*	*E. coli*	–	–
*S. aureus*	*S. aureus*	*E. coli*	–	–
*E. faecalis*	*E. faecalis*	*E. coli*	–	–
*E. coli*	*E. coli*	*S. pneumoniae*	–	–
*S. epidermidis*	*S. epidermidis*	*E. coli*	–	–
*E. coli*	*E. coli*	*S. hominis*	–	–
*K. pneumoniae*	*K. pneumoniae*	*S. pneumoniae*	–	–
*E. coli*	*E. coli*	*S. pneumoniae*	–	–
*K. pneumoniae*	*K. pneumoniae*	*S. pneumoniae*	–	–
*K. pneumoniae*	*K. pneumoniae*	*P. aeruginosa*	–	–
*E. coli*	*E. coli*	*S. epidermidis*	–	–
*E. coli*	*E. coli*	*E. faecalis*	–	–
*E. coli*	*E. coli*	*A. baumannii*	–	–
*V. streptococci*	*V. streptococci*	*E. coli*	–	–
*K. pneumoniae*	*K. pneumoniae*	*E. coli*	–	–
*E. coli*	*E. coli*	*S. epidermidis*	–	–
*E. coli*	*E. coli*	*A. baumannii*	–	–
*E. coli*	*E. coli*	*K. pneumoniae*	–	–
*E. coli*	*E. coli*	*E. faecium*	–	–
*S. hominis*	*S. hominis*	*E. coli*	–	–
*P. mirabilis*	*P. mirabilis*	*S. pneumoniae*	–	–
*E. coli*	*E. coli*	*K. pneumoniae*	–	–
*S. hominis*	*S. hominis*	*S. epidermidis*	–	–
*P. aeruginosa*	*P. aeruginosa*	*S. pneumoniae*	–	–
*K. pneumoniae*	*K. pneumoniae*	*S. pneumoniae*	–	–
*S. hominis*	*S. hominis*	*K. pneumoniae*	–	–
*S. aureus*	*S. aureus*	*E. coli*	–	–
*E. faecalis*	*E. faecalis*	*P. mirabilis*	–	–
*C. albicans*	*C. albicans*	*E. faecium*	–	–
*K. pneumoniae*	*K. pneumoniae*	*S. maltophilia*	–	–
*A. baumannii*	*A. baumannii*	*P. mirabilis*	–	–
*E. faecalis*	*E. faecalis*	*E. cloacae*	–	–
*S. aureus*	*S. aureus*	*A. baumannii*	–	–
*S. hominis*	*S. hominis*	*E. cloacae*	–	–
*S. aureus*	*S. aureus*	*E. cloacae*	–	–
*S. hominis*	*S. hominis*	*S. epidermidis*	–	–
*C. albicans*	*C. albicans*	*S. pneumoniae*	–	–
*E. faecalis*	*E. faecalis*	*E. coli*	–	–
*S. hominis*	*S. hominis*	*S. epidermidis*	–	–
*P. aeruginosa*	*P. aeruginosa*	*E. coli*	–	–
*S. aureus*	*S. aureus*	*E. coli*	–	–
*S. hominis*	*S. hominis*	*E. coli*	–	–
*S. hominis*	*S. hominis*	*E. aerogenes*	–	–
*S. epidermidis*	*S. epidermidis*	*E. cloacae*	–	–
*E. faecalis*	*E. faecalis*	*E. coli*	–	–
*E. aerogenes*	*E. aerogenes*	*K. pneumoniae*	–	–
*S. agalactiae*	*S. agalactiae*	*K. pneumoniae*	–	–
*C. parapsilosis*	*C. parapsilosis*	*S. marcescens*	–	–
*C. albicans*	*C. albicans*	*A. baumannii*	–	–
*S. hominis*	*S. hominis*	*C. albicans*	–	–
*C. parapsilosis*	*C. parapsilosis*	*C. glabrata*	–	–
*E. faecium*	*E. faecium*	*E. faecium*	–	–
*C. tropicalis*	*C. tropicalis*	*E. faecalis*	–	–
*V. streptococci*	*V. streptococci*	*A. baumannii*	–	–
*C. albicans*	*C. albicans*	*E. cloacae*	–	–
*E. cloacae*	*E. cloacae*	*E. faecium*	–	–
*E. cloacae*	*E. cloacae*	*S. aureus*	–	–
*C. parapsilosis*	*C. parapsilosis*	*E. faecium*	*S. maltophilia*	–
*E. faecium*	*E. faecium*	*S. maltophilia*	*S. agalactiae*	*S. haemolyticus*

^*a*^ "–" indicates not applicable.

### Detection performance of PCR-QDFA in polymicrobial growth

Among the 2,619 samples recruited, 50 cases were identified as polymicrobial in blood culture bottles, including 48 double pathogens and 2 triple pathogens (Table 4). *K. pneumoniae*, *E. coli,* and *E. faecium* were the most isolated microorganisms. The results of blood culture and the PCR-QDFA assay for the identification of polymicrobial infection are shown in Table 4. Overall, the PCR-QDFA method successfully identified 105 microorganisms from the blood culture bottles. The results of the PCR-QDFA assay were completely consistent with those of blood culture in 47 cases. In the remaining three samples, PCR-QDFA confirmed blood culture results; the assay identified an additional bacterium in each of these samples, namely *E. faecalis*, *S. epidermidis*, and *A. baumannii*, respectively. Subsequent validation by third-party Sanger sequencing confirmed the presence of *E. faecalis*, *S. epidermidis*, and *A. baumannii* in these three samples.

**TABLE 4:** Direct identification of bacteria and yeasts in 50 polymicrobial blood culture bottles by the PCR-QDFA assay

Identification	Detection by^*a*^	
	Blood culture	PCR-QDFA
*E. coli*, *K. pneumoniae*	1,1	1,1
*E. coli*, *K. pneumoniae*	1,1	1,1
*E. coli*, *K. pneumoniae*	1,1	1,1
*E. coli*, *K. pneumoniae*	1,1	1,1
*E. coli*, *K. pneumoniae*	1,1	1,1
*E. coli*, *K. pneumoniae*	1,1	1,1
*E. coli*, *K. pneumoniae*	1,1	1,1
*E. coli*, *K. pneumoniae*	1,1	1,1
*E. coli*, *K. pneumoniae*	1,1	1,1
*E. faecalis, E. faecium*	1,1	1,1
*E. faecalis, E. faecium*	1,1	1,1
*E. cloacae, E. faecium*	1,1	1,1
*E. cloacae, E. faecium*	1,1	1,1
*E. faecium, E. coli*	1,1	1,1
*E. faecium, E. coli*	1,1	1,1
*S. hominis, E. faecium*	1,1	1,1
*S. hominis, E. faecium*	1,1	1,1
*E. faecium, A. baumannii*	1,1	1,1
*E. faecium, A. baumannii*	1,1	1,1
*E. faecalis*, *K. pneumoniae*	1,1	1,1
*E. faecalis, K. pneumoniae*	1,1	1,1
*E. faecalis*, *K. pneumoniae*	1,1	1,1
*E. faecalis*, *K. pneumoniae*	1,1	1,1
*K. pneumoniae, E. faecium*	1,1	1,1
*K. pneumoniae*, *E. faecium*	1,1	1,1
*E. coli*, *C. albicans*	1,1	1,1
*E. coli*, *E. cloacae*	1,1	1,1
*K. oxytoca*, *E. coli*	1,1	1,1
*E. coli*, *P. mirabilis*	1,1	1,1
*E. faecalis, K. oxytoca*	1,1	1,1
*E. faecalis, C. albicans*	1,1	1,1
*C. glabrata, C. albicans*	1,1	1,1
*E. coli, V. streptococci*	1,1	1,1
*E. faecium, C. albicans*	1,1	1,1
*E. faecium, P. aeruginosa*	1,1	1,1
*S. aureus, S. epidermidis*	1,1	1,1
*E. faecium, S. maltophilia*	1,1	1,1
*K. pneumoniae, P. mirabilis*	1,1	1,1
*C. albicans, V. streptococci*	1,1	1,1
*K. pneumoniae, C. tropicalis*	1,1	1,1
*P. aeruginosa*, *K. pneumoniae*	1,1	1,1
*A. baumannii, K. pneumoniae*	1,1	1,1
*A. baumannii, S. epidermidis*	1,1	1,1
*A. baumannii, P. aeruginosa*	1,1	1,1
*K. pneumoniae*, *E. aerogenes*	1,1	1,1
*C. albicans, C. tropicalis, E. faecium*	1,1,1	1,1,1
*K. pneumoniae*, *P. mirabilis, E. coli*	1,1,1	1,1,1
*K. pneumoniae*, *E. coli*, *E. faecalis*	1,1,0	1,1,1
*E. faecium, S. haemolyticus, S. epidermidis*	1,1,0	1,1,1
*E. faecium, E. faecalis, A. baumannii*	1,1,0	1,1,1

^*a*^ 1, detection of a microorganism in the PCR-QDFA panel; 0, failure to detect.

### Overall performance of PCR-QDFA in pathogen identification

The performance of PCR-QDFA in identifying each pathogen compared to blood culture identification methods is shown in Table 1. For all tested pathogens, PCR-QDFA identified 100% (2,195/2,195) of the pathogens identified by blood culture, with a sensitivity of 100% and specificity of 90.34% (430/476), achieving an overall agreement of 98.28% (2,625/2,671). For the 12 gram-negative bacteria, the results of PCR-QDFA were consistent with blood culture in 1,184/1,184 cases, demonstrating 100% sensitivity and 93.52% (1,342/1,435) specificity. Similarly, for the 11 gram-positive bacteria, PCR-QDFA showed a sensitivity of 100% (876/876) and specificity of 96.27% (1,678/1,743). Notably, PCR-QDFA exhibited a sensitivity of 100% (135/135) and a specificity of 99.80% (2,479/2,484) for the four *Candida* species.

## RESULTS AND DISCUSSION

A total of 2,619 blood culture bottles were recruited from different patients in this study. Parallel detection and comparative studies were conducted with the registered VITEK 2 Identification Card (bioMérieux Diagnostics Products [Shanghai] Co., Ltd.) to evaluate the equivalence of the blood pathogen nucleic acid detection kit (PCR-QDFA) and blood culture. In this experiment, the kappa values of the PCR-QDFA for the determination of 27 types of bacteria collected were all between 0.81 and 1.00, indicating that the diagnostic results of the determination were almost completely consistent with blood culture, suggesting that PCR-QDFA has excellent testing efficacy for both monomicrobial bloodstream infections and polymicrobial bloodstream infections.

Compared with previous similar studies (7, 8), our study introduced Sanger sequencing as an additional validation method. Blood culture, while widely accepted, has inherent limitations such as reduced sensitivity in patients receiving antimicrobial therapy, prolonged turnaround time, and the test was not validated for non-cultivable organisms. Using blood culture alone as the “gold standard” may therefore introduce certain bias. By incorporating Sanger sequencing, we were able to provide molecular-level confirmation of PCR results, which strengthened the reliability of the diagnostic evaluation. This three-way verification framework (PCR, blood culture, and Sanger sequencing) enhanced the rigor of our analysis and helped clarify potential discrepancies between PCR and culture results, for instance, those related to contamination, PCR inhibition, or the presence of low-abundance pathogens. Thus, our approach provides more robust evidence to support the clinical application of PCR in bloodstream infection diagnosis.

One discordant case was observed in which *Burkholderia cepacia* was positive by the investigational assay but negative by both blood culture and Sanger sequencing, potentially due to sample contamination, reagent mix-up, or pipetting errors. Six cases of *Staphylococcus simulans* were misidentified as *Staphylococcus haemolyticus*, likely because the PCR-QDFA panel lacked *S. simulans*-specific targets, and the high genetic similarity between these species led to primer cross-reactivity. Future studies should focus on expanding the spectrum of detectable pathogens.

Polymicrobial BSIs (5%–38% incidence) carry two- to threefold higher mortality (21%–63%) and prolonged hospitalization versus monomicrobial BSIs, underscoring the need for accurate detection (9). Current molecular diagnostics include sequencing, MALDI-TOF mass spectrometry, fluorescence *in situ* hybridization, and PCR. While high-throughput sequencing and digital PCR offer optimal polymicrobial BSI detection (10), the former suffers from complex workflows, high costs, and prolonged turnaround (9, 11, 12), and the latter lacks clinically approved assays for BSIs (13, 14).

The PCR-QDFA assay circumvents this limitation, conferring accelerated turnaround times and superior polymicrobial detection capability. The PCR-QDFA assay addresses this limitation by offering shorter turnaround times and enhanced capability for polymicrobial detection. Additionally, the PCR-QDFA assay addresses this limitation by offering shorter turnaround times and enhanced capability for polymicrobial detection. Compared to sequencing, it simplifies data analysis and reduces costs, while covering a broader range of bacterial targets than digital PCR. The assay incorporates quantum dots, which provide size-tunable emission, narrow spectral bands, high brightness, and improved photostability. Furthermore, signal amplification via streptavidin–biotin interaction increases sensitivity for trace pathogen detection (7, 15). The use of streptavidin-coated quantum dots also improves specificity by minimizing nonspecific binding. These attributes suggest that PCR-QDFA could become a valuable tool for BSI diagnosis, particularly in time-critical sepsis management, rapid identification of polymicrobial infections, and clinical settings requiring high sensitivity and specificity, such as intensive care units or resource-limited environments will likely have access to this type of technology.

Our study also has a few limitations. As a PCR-based method, it is restricted to detecting pre-targeted bacteria and may miss unusual or unexpected pathogens. Compared with other PCR-based detection systems, the number of pathogens it can identify is relatively small, and no resistance genes are detected (16, 17), which limits its usefulness for guiding antimicrobial therapy. Future work should aim to improve the detection of resistant genes and broaden the panel of detectable pathogens, which would further enhance clinical utility.

In summary, PCR-QDFA provides rapid, accurate detection of common bloodstream pathogens and demonstrates strong performance in both mono- and polymicrobial infections. These findings support its potential role as a complementary tool to conventional culture, particularly in settings where rapid, actionable results are critical for patient management.

## Data Availability

The data that support the findings of this study are available from the corresponding author upon reasonable request.

## SUPPLEMENTAL MATERIAL

Supplemental material spectrum.03845-25-s0001.docx Primer and probe information.

ASM does not own the copyrights to Supplemental Material that may be linked to, or accessed through, an article. The authors have granted ASM a non-exclusive, world-wide license to publish the Supplemental Material files. Please contact the corresponding author directly for reuse.
