# Supplementary material for: A multicenter clinical evaluation of polymerase chain reaction coupled with quantum dot fluorescence analysis in pathogen detection for patients with suspected bloodstream infections
Source: Microbiol Spectr. 2026 Jul 21;14(9):e03845-25. doi: 10.1128/spectrum.03845-25 (PMC13532286; doi:10.1128/spectrum.03845-25)
Supplement: Supplementary file 1 [file spectrum.03845-25-s0001.docx]

**Supplementary Primer and Probe Information**

1. A quantum dot nucleic acid detection kit for bloodstream infection pathogens, characterized in that the kit comprises a detection membrane strip, a fluorescence detection solution, and reaction solutions, wherein:

The detection membrane strip comprises a nylon membrane and capture probes immobilized on the nylon membrane;

The fluorescence detection solution comprises quantum dots surface-conjugated with streptavidin for labeling the capture probes;

The reaction solutions comprise: Reaction Solution I, Reaction Solution II, Reaction Solution III, and Reaction Solution IV;

Reaction Solution I contains the following detection primers:

Primer JSF23F1: GAGATTTCCGAATGGGGAAACCCA;

Primer LG23F1: GCGATGTCCGAATGGGGAAACCCA;

Primer BK23F1: ACGATTTCCGAATGGGGCAACCCA;

Primer CS23F1: AAGATATCCGAATGGGGAAACCCA;

Primer NZ23F1: GTCAGAGGCGATGAAGGGCG;

Primer Z23F1: GTAATGTCCGAATGGGGAAACCCA;

Primer BMTLF1: CGGCAAATATCCTTTGATCCG;

Primer JSFZNBCY23R1: CCTTTCCCTCACGGTAC;

Primer LG23R1: TTCGCCTTTCCTTCACAGTACTG;

Primer HBBF1: TATGGTTGGGATAAGGCTGG;

Primer HBBR1: CGAGCTTAGTGATACTTGTG;

Reaction Solution II contains the following detection primers:

Primer TUF1: GAATTGGTTGAAATGGAAAT;

Primer TUR1: CCACGGTCGATACGTCCTGA;

Primer CNSF1: GAATTGGTTGAAATGGAAAT;

Primer CNSR1: CCACGGTCGATACGTCCTGA;

Primer FKYBIF1: TGCTCACACAGATTGTCTG;

Primer YGR1: CAGGCGCTCTCCCAGCTGAG;

Primer FKR1: ATCCAGATTTTTAAAGAGCA;

Primer QYR1: CTTGTTCCAGATTGTTAAAGAGCA;

Primer PTR1: AATATACCTCGGTGATATATTG;

Primer HBBF1: TATGGTTGGGATAAGGCTGG;

Primer HBBR1: CGAGCTTAGTGATACTTGTG;

Reaction Solution III contains the following detection primers:

Primer VANF1: TAGGCTGCGATATTCAAAGCTC;

Primer VANR1: AACAGCCTGCTCAATTAAGATTTT;

Primer VANR2: CCGACCTCACAGCCCGAAA;

Primer MEF1: CGTTCAATTTAATTTTGTTAAA;

Primer MER1: TTTTAGATACATTCTTTGGAAC;

Primer ECOF1: GTCTGCAATTGCCACCACTG;

Primer ECOR1: GCCGAATCGCTGCTGGAAG;

Primer ECOF2: CATATCTTCTTCCAGATTGC;

Primer ECOR2: GCTGTTTGGCTATGAGGAAG;

Primer ITSF1: GGTTTCCGTAGGTGAAC;

Primer ITSR1: GCATCCCACGGGCTCTCACC;

Primer CQF1: GGCGGTCTGCACGGCGTAG;

Primer CQR1: CCAGGCTTGGCCAGAAAC;

Primer HBBF1: TATGGTTGGGATAAGGCTGG (SEQ ID No. 10);

Primer HBBR1: CGAGCTTAGTGATACTTGTG (SEQ ID No. 11);

Reaction Solution IV is Hotstart Taq DNA polymerase.

2. The quantum dot nucleic acid detection kit for bloodstream infection pathogens according to claim 1, characterized in that the capture probes comprise:

Probe BPP1: CAGATGCTGGTAAATTACCWTG;

Probe CQP1: TATCCAACGCGATAACAAAGT;

Probe VAP1: CGTTCAGGCTCATCCTTCGG;

Probe VBP1: TCAGGTTCGTCCTTTGGCGTAA;

Probe MEP1: AAAGCATACATATTGAAAATTT;

Probe RXP1: GACGTAGAAGTWGTTGAAGGTG;

Probe FSPP1: TGAAGTAGAAAAAGACGGTT;

Probe RXXP1: GTTGAAGTTRTTGATGGTG;

Probe HNP1: ATTACAGGTCGTGGTACAGTC;

Probe WRP1: GAACCAGATGCTGGTAAATTAC;

Probe FYLP1: TCAGCACTTAAAGCTCTTGAAG;

Probe CLP1: GTTCAGCTCTTAAAGCCCTTGAAG;

Probe CAP1: TTGCGCCCTCTGGTATTCC;

Probe CGP1: GTTGTGTTTGGTAGTGAGTG;

Probe CPP1: AAATCAGGTAGGACTACCCG;

Probe CTP1: AACTTTCAACAACGGATCTCTT;

Probe CKP1: GGACGACGTGTAAAGAGCG;

Probe ECOP1: GGTCATTAGCGCCACTCACTGCA;

Probe SAP1: TAGCATATCAGAAGGCACACC;

Probe SCP1: AGCTGATTAGAGGTAGAC;

Probe FCP1: AGCACATTCGAGGTAGAC;

Probe TLP1: GTGTCACGTAAGTGACGCG;

Probe BMP1: ATGTATACTTTGTATACGTG;

Probe BKP1: AGTGCGGCCATAGCAGGTG;

Probe ZSP1: TCGCGCAGTAAAGGGTGAT;

Probe NZP1: TTTCGACACACTATCATGTCA;

Probe CSP1: GTCGCAGGGTACAGGGTGAT;

Probe LGP1: GGAAGCACAATCAAAGAGG;

Probe YGP1: CACATACWTAAGTATGCTTCGT;

Probe FKP1: AGTGCTCACACAGATTGTCT;

Probe PTP1: TTGTRAAATCACTCGTTTACTGA;

Probe QYP1: GTTAAAGTCCAATCAGGCCTA.

3. The quantum dot nucleic acid detection kit for bloodstream infection pathogens according to claim 2, characterized in that the detection membrane strip further comprises an internal control probe for monitoring sample nucleic acid extraction and amplification: ICP1: TTTGCTAATCATGTTCATACC.

4. The quantum dot nucleic acid detection kit for bloodstream infection pathogens according to claim 2, characterized in that the capture probes are oligonucleotide single-stranded DNA, wherein the 3′ end or 5′ end of the oligonucleotide single-stranded DNA is labeled with an amino group, and a spacer arm is linked between the oligonucleotide single-stranded DNA and the amino group; the spacer arm is one of or a combination of a fatty acid C(n) chain and oligo dT(n), wherein n in the fatty acid C(n) chain is an integer from 1 to 12, and n in oligo dT(n) is an integer from 1 to 30.

5. The quantum dot nucleic acid detection kit for bloodstream infection pathogens according to claim 1, characterized in that the 5′ ends of the reverse primers among the detection primers are modified with a biotin label, and a spacer arm is linked between the detection primers and the biotin; the spacer arm is one of or a combination of a fatty acid C(n) chain and oligo dT(n), wherein n in the fatty acid C(n) chain is an integer from 1 to 12, and n in oligo dT(n) is an integer from 1 to 30.

6. The quantum dot nucleic acid detection kit for bloodstream infection pathogens according to claim 1, characterized in that the excitation wavelength of the quantum dots is 200–500 nm, the emission wavelength of the quantum dots is 400–700 nm, and the particle size of the quantum dots is 1–200 nm.

7. The quantum dot nucleic acid detection kit for bloodstream infection pathogens according to claim 1, characterized in that the quantum dots are CdSe/ZnS.

**The above content can be found at the China National Intellectual Property Administration (CNIPA).**
